# Who can benefit from postmastectomy radiotherapy among HR+/HER2- T1-2 N1M0 breast cancer patients? An explainable machine learning mortality prediction based approach

**DOI:** 10.3389/fendo.2024.1326009

**Published:** 2024-02-02

**Authors:** Long Jin, Qifan Zhao, Shenbo Fu, Yuan Zhang, Shuhan Wu, Xiao Li, Fei Cao

**Affiliations:** ^1^ Department of Radiation Oncology, Shaanxi Provincial People’s Hospital, Xi’an, China; ^2^ Department of Computer Science, The University of Hong Kong, Hong Kong, Hong Kong SAR, China; ^3^ Department of Radiation Oncology, Shaanxi Provincial Cancer Hospital, Xi’an, China; ^4^ Department of Oncology, Shaanxi Provincial People’s Hospital, Xi’an, China; ^5^ Department of Surgical Oncology, Shaanxi Provincial People’s Hospital, Xi’an, China; ^6^ Internal Medicine, St. Luke's Hospital, Chesterfield, MO, United States

**Keywords:** HR+/HER2-, subgroup analysis, machine learning, PMRT, breast cancer

## Abstract

**Objective:**

The necessity of postmastectomy radiotherapy(PMRT) for patients with HR+/HER2 T1-2 N1M0 breast cancer remains controversial. We want to use explainable machine learning to learn the feature importance of the patients and identify the subgroup of the patients who may benefit from the PMRT. Additionally, develop tools to provide guidance to the doctors.

**Methods:**

In this study, we trained and validated 2 machine learning survival models: deep learning neural network and Cox proportional hazard model. The training dataset consisted of 35,347 patients with HR+/HER2- T1-2 N1M0 breast cancer who received mastectomies from the SEER database from 2013 to 2018. The performance of survival models were assessed using a concordance index (c-index).Then we did subgroup analysis to identify the subgroup who could benefit from PMRT. We also analyzed the global feature importance for the model and individual feature importance for individual survival prediction. Finally, we developed a Cloud-based recommendation system for PMRT to visualize the survival curve of each treatment plan and deployed it on the Internet.

**Results:**

A total of 35,347 patients were included in this study. We identified that radiotherapy improved the OS in patients with tumor size >14mm and age older than 54: 5-year OS rates of 91.9 versus 87.2% (radio vs. nonradio, P <0.001) and cohort with tumor size >14mm and grade worse than well-differentiated, 5-year OS rates of 90.8 versus 82.3% (radio vs. nonradio, P <0.001).The deep learning network performed more stably and accurately in predicting patients survival than the random survival forest and Cox proportional hazard model on the internal test dataset (C-index=0.776 vs 0.641) and in the external validation(C-index=0.769 vs 0.650).Besides, the deep learning model identified several key factors that significantly influence patient survival, including tumor size, examined regional nodes, age at 45-49 years old and positive regional nodes (PRN).

**Conclusion:**

Patients with tumor size >14mm and age older than 54 and cohort with tumor size >14mm and grade worse than well-differentiated could benefit from the PMRT. The deep learning network performed more stably and accurately in predicting patients survival than Cox proportional hazard model on the internal test. Besides, tumor size, examined regional nodes, age at 45-49 years old and PRN are the most significant factors to the overall survival (OS).

## 1 Introduction

According to the latest GLOBOCAN 2020 estimates from the International Agency for Research on Cancer, breast cancer has emerged as the most common malignant tumor globally, surpassing lung cancer (1). There are 2.26 million new cases of breast cancer reported globally. Among three major subtypes based on hormone receptor and epidermal growth factor receptor status, HR+/HER2- breast cancer is the most common subtype, accounting for one-third of all breast cancers (2). Furthermore, survival rates vary among different breast cancer subtypes. The most favorable survival pattern was observed among women with the HR+/HER2- subtype with a survival rate of 92.5% at 4 years (3).

The necessity of PMRT for patients with early-stage breast cancer remains a topic of debate. While radiation therapy has been the standard adjuvant therapy for patients with tumors >5 cm or lymph node metastasis >4 (4), its role in patients with early-stage breast cancer with tumors <5 cm (T1-2) or with one to three lymph node metastases (N1) is controversial (5). Some studies support the use of PMRT in these patient populations. The meta-analysis of the Early Breast Cancer Trialists Collaborative Group suggested that PMRT can reduce the recurrence rate and breast cancer mortality in patients with 1-3 positive lymph nodes after mastectomy and axillary lymph node dissection. However, it did not show a significant impact on OS (6). The Consensus Discussion at St. Gallen/Vienna 2019 indicated varying opinions, 29% of the experts indicated varying opinions with risk factors such as triple-negative cancer or positive margins (7). Organizations like ASCO, ASTRO and SSO also support PMRT for T1-2N1 patients to reduce cancer mortality (8). On the other hand, there are some different views. The 10-year follow-up results of the BCIRG-005 trial suggested that PMRT improved local-regional control but had no impact on OS in T1-3N1 breast cancer patients (9). Earlier studies, such as BIG 02/98 and BCIRG001, also found postoperative radiotherapy has no significant effect on OS or relapse-free survival for patients with T1-T2 N1 disease receiving standard adjuvant systemic therapy (10).

However, these trials did not include some important clinicopathological factors, such as ER and/or PR and HER2 status. In certain molecular subtypes of breast cancer, the potential benefit of radiation therapy may be outweighed by its associated toxicities. For instance, a study retrospectively analyzed 16,521 patients with breast cancer T1-2N1 from 2010 to 2014, the survival analysis showed that PMRT was beneficial for patients with Luminal A type, resulting in a 24.1% reduction in the risk of death. However, patients with Luminal B, Her-2 positive, and triple-negative patients failed to benefit from radiotherapy. It is worth noting that another study by Liu et al. did get the opposite conclusion that the Luminal A type may not benefit from postoperative radiotherapy (11). As breast cancer research progresses, oncologists are facing information management challenges. While computational systems have been developed to assist with clinical decision-making, they have not yet been adopted in clinical practice (12). Therefore, we combined patient’s clinicopathological characteristics and utilized deep learning to develop a model for HR+/HER2-T1- 2N1M0 breast cancer patients. This model provides recommendations and incorporates an explainable module that explains the log hazard rate prediction from the model (13). Besides, many research projects consider deep learning model as back boxes, lacking in transparency and trustworthiness. To address this issue, we applied the technology of explainable AI to establish a communication bridge between humans and the model (14). This enables clinicians to understand the recommendation provided by the deep learning model (15). This facilitates the understanding of why the model provides specific decisions regarding PMRT

## 2 Method

### 2.1 Eligibility criteria and patient information

Based on the November 2020 submission, we selected 35347 medical cases as the training cohort from the database: Incidence - SEER Research Plus Data, 18 Registries, Nov 2020 Sub (2000-2018) - Linked To County Attributes - Total U.S., 1969-2019 Counties, National Cancer Institute, DCCPS, Surveillance Research Program, released April 2021. We included the cases that met the following criteria, (1) female patients diagnosed pathologically with Luminal A or Luminal B T1-2 N1 M0 breast cancer between January 2013 and December 2018, (2) the existence of one malignant lesion. Conversely, we excluded cases that met the following criteria, (1) patients who did not undergo mastectomy as part of the course of treatment, (2) patients with uncertain or missing tumor size data. These included demographic information (age and marital status at diagnosis), breast-cancer-related attributes (TNM stage, histology type, primary site, tumor size, the number of regional nodes examined, grade, ER status, PR status, the number of PRN and tumor laterality), and treatment details (surgery of primary site, radiation, and chemotherapy). The primary outcomes of interest were patient survival time and mortality indicator.

For the external validation cohort, we randomly collected data from 145 Luminal A or Luminal B T1-2 N1 M0 breast cancer patients from January 2013 to December 2018 at Shaanxi Provincial People’s Hospital in China.

### 2.2 Explainable machine learning survival model design

**Figure 1:**
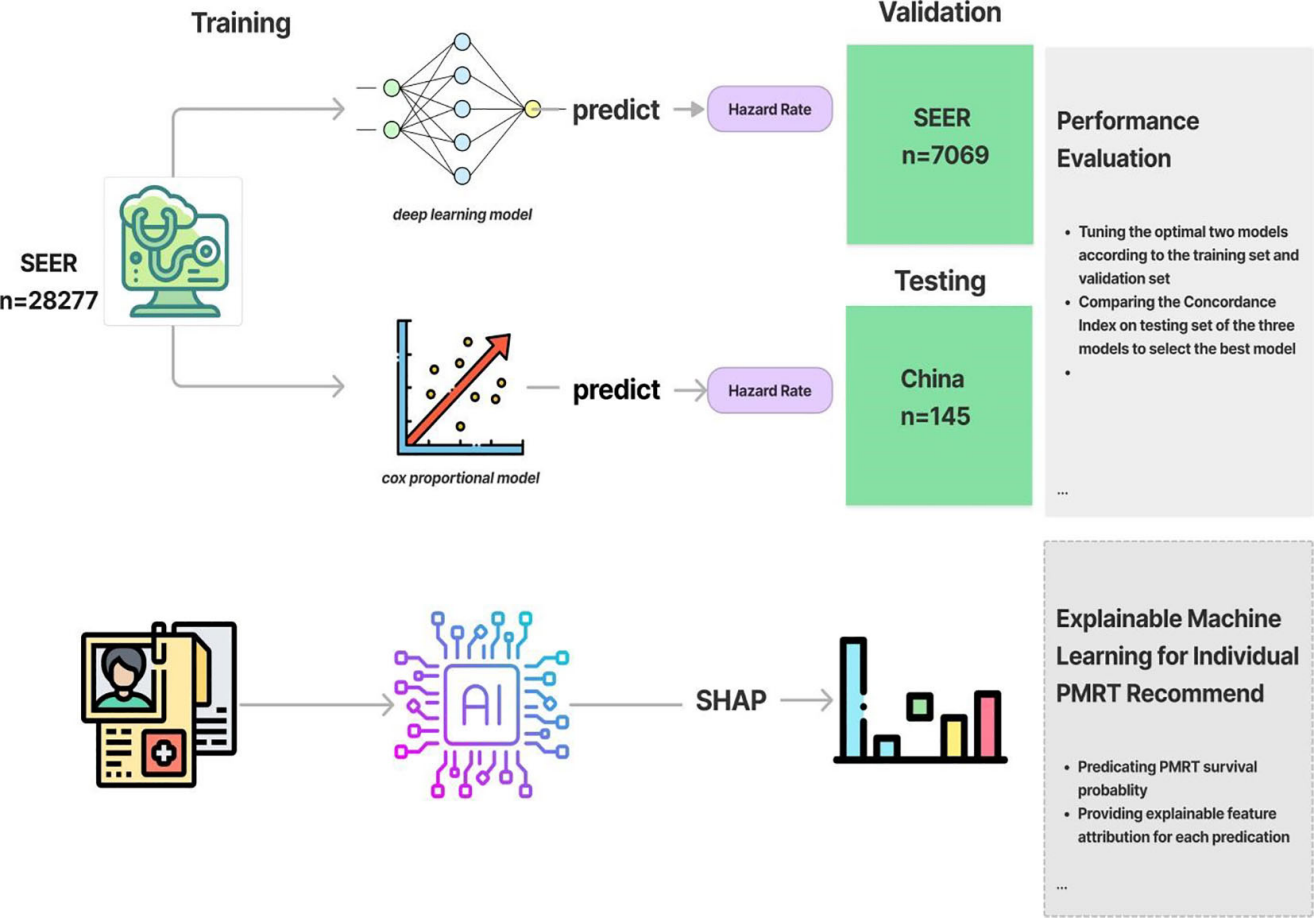
Diagram of the training and recommendation procedure.

In this part, we’ll train and examine the performance of two machine learning models that perform survival prediction. The two algorithms are trying to fit the relationship between covariates and the log hazard of an individual. The model’s architecture consists of an input layer that takes the patient’s baseline data, followed by fully connected hidden layers interspersed with dropout layers. The output of the network is the log hazard. The Rectified Linear Unit (ReLU) was used as activation function. To update the model’s parameters over numerous epochs, we utilized the Adam algorithm for gradient descent(**?**). We applied the random search to the log space of the learning rate in [0.001, 0.1], the dropout rate in [0.5-0.8], the number of hidden layers in [1,10] and the number of nodes in each hidden layer in [10,80]. Next, we trained the Penalized Cox Proportional hazard model and tuned the hyperparameter by using random search method, specifically, the penalizer in [0.001,1] and the learning rate in [0.001,1]. Regarding the explainable module, Shapley value was used on each clinical feature value to understand their individual contributions to the neural network’s predictions. Initially for individual predictions, we calculated each feature’s shapley value and generate a waterfall plot visualize how the neural network arrived at its predictions based on each clinical feature value of an individual feature of the breast cancer patient. To calculate the global feature importance, we average the absolute Shapley values per feature across the large training data. Then the features were sorted by decreasing importance and plotted (**Figure 1**).

### 2.3 Cloud-based PMRT recommender system deployment

To use the optimal model, we input feature values based on the patient’s demographic features, morphology, extent of breast cancer, therapy and the stage information. As for PMRT recommendation, we predict the hazard rate under two treatments (with PMRT and without PMRT). Then, we subsequently derive the two 5-year survival functions. To enhance user experience, we have also implemented UI code that visualizes the predicted survival functions using a line race chart.

### 2.4 Computation software

The models are trained with Python version 3.9; the deep learning approach is trained with PyTorch version 1.11.0; the penalized cox proportional hazard model is trained with PySurvival version 0.1.2. The Vue.js javascript framework and the Vuetify Material Design component framework were used to develop the front user interface (UI) of the adjuvant therapy recommender system. The backend code of the web application is implemented using the Django REST framework. The recommender system may be accessed using a web browser on Tencent Cloud.

## 3 Results

### 3.1 Patient baseline characteristics

In this study, we included 35,491 HR+/HER2- T1-2 N1 M0 Breast cancer patients who underwent surgery based on the inclusion criteria. The cohort was divided into a training set consisting of 35,246 patients from the SEER database, and a test set of 145 patients from the China Database for model testing. **Table 1** shows the baseline medical characteristics for the two cohorts. In the SEER cohort, infiltrating duct carcinoma accounts for the majority of patient histological types (77.55%). Lobular carcinoma was the second most common histological type, accounting for 8.74% of cases. In terms of the breast molecular subtype, 86.86% of patients had Luminal A breast cancer, while the remaining 13.13% had Luminal B molecular subtypes. Approximately 55.77% of patients underwent beam radiation and 61.63% received chemotherapy as part of the adjuvant regimen.

**Table 1:** Main baseline clinical characteristics of patients.

Characteristic	Data set, No. (%)	
	Training	Testing ($)
Age		
85+ years	705(1.99)	0
80-84 years	1290(3.65)	1(0.69)
75-79 years	1932(5.46)	7(4.83)
70-74 years	2986(8.44)	12(8.27)
65-69 years	4225(11.95)	11(7.58)
60-64 years	4770(13.49)	13(8.96)
55-59 years	4716(13.34)	21(14.48)
50-54 years	4888(13.82)	19(13.10)
45-49 years	4496(12.71)	21(14.48)
40-44 years	3013(8.52)	23(15.86)
35-39 years	1444(4.08)	8(5.51)
30-34 years	637(1.80)	5(3.44)
25-29 years	202(0.57)	3(2.06)
20-24 years	41(0.11)	1(0.68)
15-19 years	1(0.003)	0
Histologic type		
Infiltrating duct carcinoma, NOS	27410(77.55)	143(98.62)
Lobular carcinoma, NOS	3090(8.74)	1(0.68)
Infiltrating duct and lobular carcinoma	2747(7.77)	0
Infiltrating duct mixed with other types of carcinoma	990(2.80)	0
Mucinous adenocarcinoma	243(0.68)	0
Ductal carcinoma, micropapillary	229(0.64)	0
Infiltrating lobular mixed with other types of carcinoma	112(0.31)	0
Intraductal papillary adenocarcinoma with invasion	57(0.16)	0
Paget disease and infiltrating ductal carcinoma of breast	56(0.15)	0
Carcinoma, NOS	55(0.15)	0
Adenocarcinoma, NOS	51(0.14)	0
Tubular adenocarcinoma	47(0.13)	0
Cribriform carcinoma, NOS	44(0.12)	0
Medullary carcinoma, NOS	33(0.09)	46(46.00)
Apocrine adenocarcinoma	24(0.06)	0
Metaplastic carcinoma, NOS	23(0.06)	0
Papillary carcinoma, NOS	21(0.05)	0
Infiltrating ductular carcinoma	20(0.05)	0
Adenocarcinoma with mixed subtypes	14(0.03)	0
Mucin-producing adenocarcinoma	8(0.02)	0
Pleomorphic carcinoma	8(0.02)	0
Papillary adenocarcinoma, NOS	7(0.02)	0
Neuroendocrine carcinoma, NOS	7(0.02)	0
Adenocarcinoma with neuroendocrine differentiation	5(0.01)	0
Secretory carcinoma of breast	4(0.01)	0
Comedocarcinoma, NOS	4(0.01)	0
Neoplasm, malignant	3(0.008)	0
Squamous cell carcinoma, NOS	3(0.008)	0
Signet ring cell carcinoma	3(0.008)	0
Solid carcinoma, NOS	3(0.008)	0
Paget disease and intraductal carcinoma	3(0.008)	0
Polymorphous low grade adenocarcinoma	2(0.005)	0
Intracystic carcinoma, NOS	2(0.005)	0
Adenosquamous carcinoma	2(0.005)	0
Atypical medullary carcinoma	2(0.005)	0
Carcinoma with osteoclast-like giant cells	2(0.005)	0
Oxyphilic adenocarcinoma	1(0.002)	0
Intraductal papillary-mucinous carcinoma, invasive	1(0.002)	0
Carcinoma, anaplastic, NOS	1(0.002)	0
Small cell carcinoma, NOS	1(0.002)	0
Paget disease, mammary	1(0.002)	0
Acinar cell carcinoma	1(0.002)	0
Carcinoma, undifferentiated, NOS	1(0.002)	0
Large cell neuroendocrine carcinoma	1(0.002)	0
Papillary carcinoma, encapsulated	1(0.002)	0
Alveolar adenocarcinoma	1(0.002)	0
Giant cell and spindle cell carcinoma	1(0.002)	0
Adenoid cystic carcinoma	1(0.002)	0
T stage		
T1mic	42(0.11)	0
T1a	627(1.77)	1(0.68)
T1b	3162(8.94)	5(3.44)
T1c	14060(39.77)	48(33.10)
T2	17455(49.38)	91(62.75)
N stage		
N1a	24283(68.70)	35(24.13)
N1mi	8591(24.30)	13(8.96)
N1	1216(3.44)	96(66.20)
N1NOS	1126(3.18)	1(0.68)
N1c	86(0.24)	0
N1b	44(0.12)	0
M stage		
M0	35346(100.00)	145(100.00)
Breast Subtype		
Luminal A	30703(86.86)	79(54.48)
Luminal B	4643(13.13)	66(45.51)
Radiation		
Beam radiation	19715(55.77)	65(44.82)
Combination of beam with implants or isotopes	37(0.10)	0
None	13161(37.23)	71(48.96)
Radiation, NOS method or source not specified	139(0.39)	9(6.20)
Recommended, unknown if administered	1548(4.37)	0
Refused	653(1.84)	0
Radioactive implants (includes brachytherapy)	88(0.24)	0
Chemotherapy		
Yes	21786(61.63)	127(87.58)
No/Unknown	13560(38.36)	18(12.41)
Grade		
Well differentiated; Grade I	6625(18.74)	12(8.27)
Moderately differentiated; Grade II	18414(52.09)	124(85.51)
Poorly differentiated; Grade III	10240(28.97)	9(6.20)
Undifferentiated; anaplastic; Grade IV	67(0.18)	0

In the test cohort, almost all patients had infiltrating duct carcinoma as their histological type, and most of them have a moderately differentiated grade. Among this population, 54.48% had Luminal A molecular subtypes, while 45.51% had Luminal B subtypes. In terms of adjuvant therapy, most patients underwent chemotherapy, nearly half of the patients received beam radiation.

### 3.2 Survival feature importance

**Figure 2:**
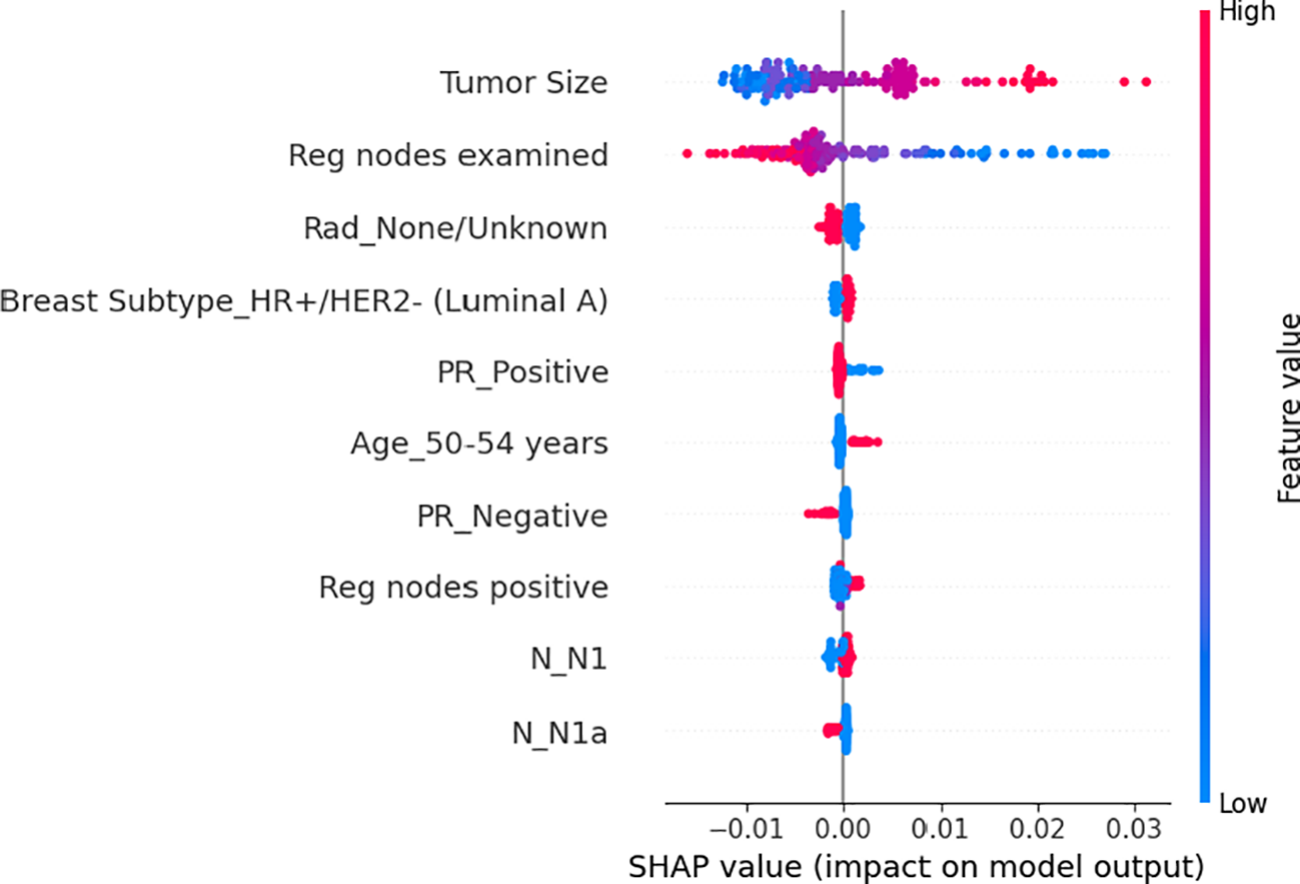
The average impact of the top 10 features from the neural network on output magnitude. The x-axis represents the mean value of shapley value, and the y-axis represents name of the input feature of the neural network.

Based on the patient data and the model, the global feature importance is determined (**Figure 2**). Initially, tumor size, examined regional node, and radiation therapy were the three most important factors for OS, which were arranged from top to bottom. The most crucial factor affecting an patient’s overall prognosis is the tumor size. Secondly, we could see how each feature’s trend related to OS. The patient’s OS will get worse as the tumor size grows since a larger tumor entails a higher hazard ratio. Similarly, an increase in PRN will have a detrimental effect on OS. However, the more examined regional nodes, the better OS. Regarding the tumor size distribution, a dense cluster exhibiting fewer tumor sizes and a small but negative SHAP value indicated that the favorable effect of smaller tumor sizes on OS is outweighed by the higher number of tumor size. Likewise, the number of the positive nodes has the same distribution.

**Figure 3:**
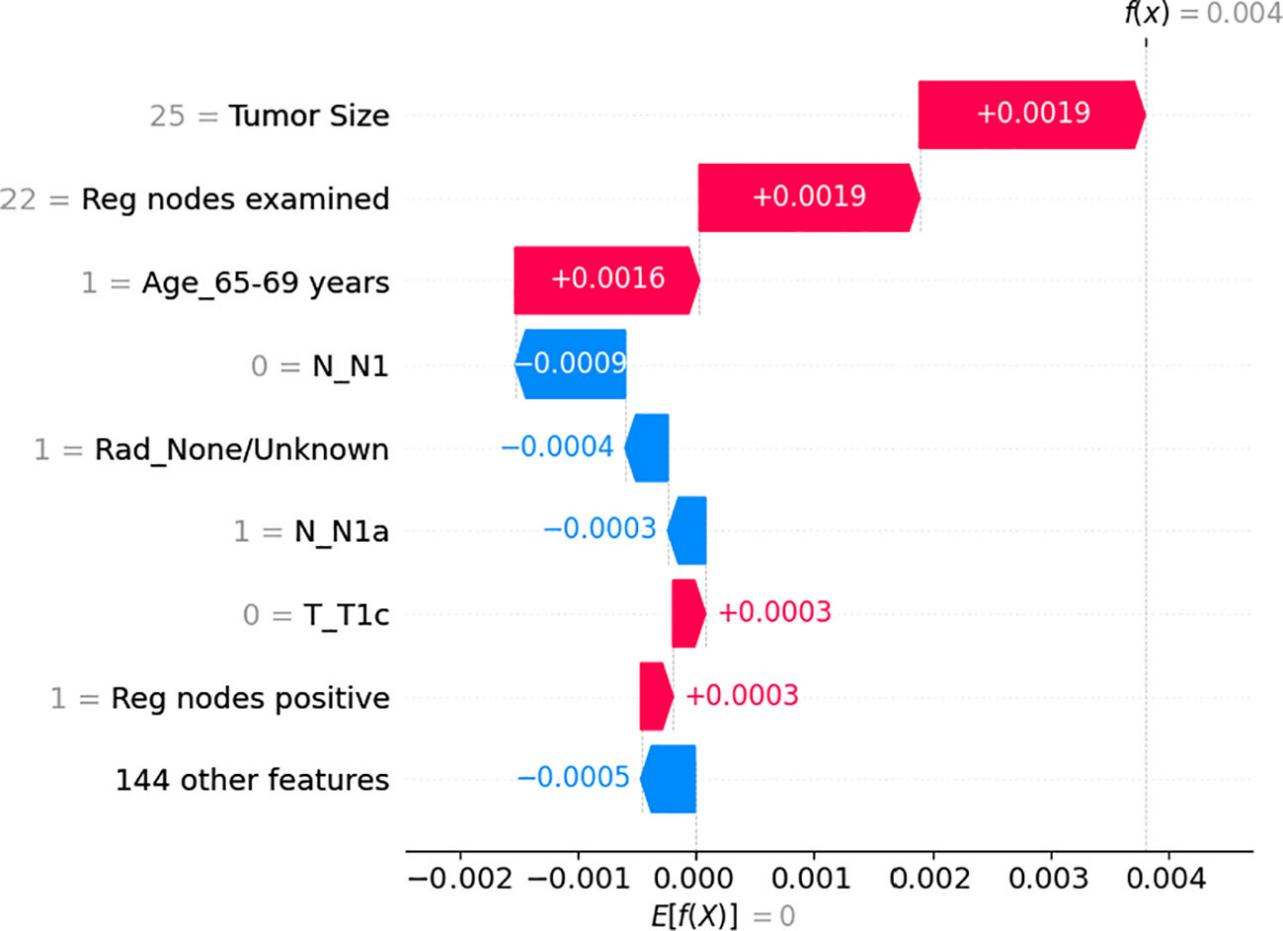
The individual feature impact of the top 8 features from the neural network on output magnitude. The x-axis represents the shapley value of the related feature and the y-axis represents name of the input feature of the neural network in risk by 0.0016 was observed due to potential additional medical issues that could interact with the breast tumor and impact treatment outcomes. Moreover, she examined 25 regional nodes, contributed to a risk increase of 0.0019. Eventually, her tumor size of 25 millimeters, which is a dangerous signal, results in a finial log hazard value of 0.0004.

Regarding individual patient feature importance (**Figure 3**), we randomly selected one patient(65-69 years old, female, T2 N1a M0, received Modified radical mastectomy) from our testing dataset to show how the model arrives at the beginning log-partial hazard(0) when it has no prior knowledge of the patient and how it predicts the patient’s outcome.(0.004). At first, all the other insignificant 144 features initially reduce the log hazard risk by 0.0005.Then, since she has 1 positive regional node and she’s T stage is not T1c, then the outcome increases by 0.0003 for each feature. Furthermore, we are filling out her N1 feature(0), radiation condition, and N1a(1), which indicate that the extent of her cancer’s spread to the lymph nodes in her armpit or other surrounding lymph nodes is not severe. Clearly, these lower her risk at 0.0003, 0.0004, and 0.0009, respectively. Considering the patient’s age range of 65 to 69 years, an increase

### 3.3 Survival analysis for subgroups

**Figure 4:**
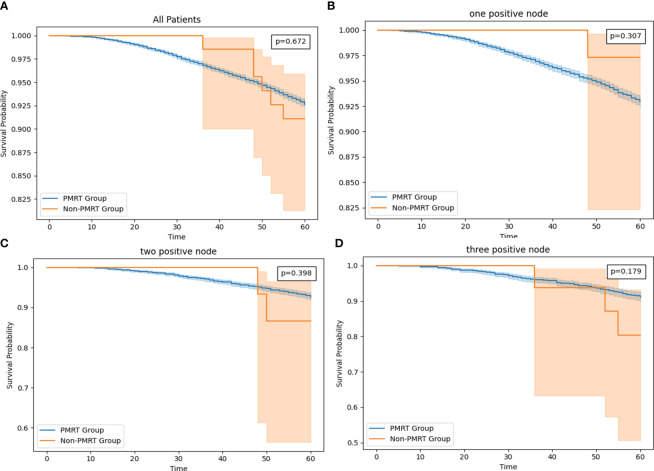
**(A)** The survival curve in all HR+/HER2- T1-2M0 breast cancer patients with PMRT and no PMRT. **(B)** The survival curve in T1-2 and one node positive HR+/HER2- T1-2M0 breast cancer patients with PMRT and no PMRT. **(C)** The survival curve in T1-2 and two positive nodes HR+/HER2- T1-2M0 breast cancer patients with PMRT and no PMRT. **(D)** The survival curve in T1-2 and one node positive HR+/HER2- breast cancer patients with PMRT and no PMRT.

**Figure 5:**
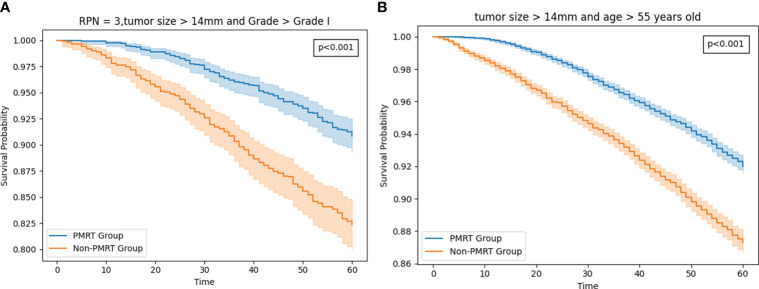
**(A)** The survival curve in Patients with tumor size >14mm and age older than 54 HR+/HER2-T1-2M0 breast cancer patients with PMRT and no PMRT. **(B)** The survival curve in T1-2 and three positive nodestumor size >14mm and grade worse than well-differentiated HR+/HER2- T1-2M0 breast cancer patients with PMRT and no PMRT.

As of December 2018, the estimated 5-year OS rates were 92.7 versus 91.1% (radio versus nonradio, P = 0.67; **Figure 4A**), and there is no significant difference of survival in all patients. Because tumor size and Age 50-54 are important features based on the result of feature importance, we perform subgroup analysis combining two variables, finding radiotherapy improved the OS in patients with tumor size >14mm and age older than 54: 5-year OS rates of 91.9 versus 87.2% (radio vs. nonradio, P <0.001; **Figure 5A**).

The more RPN may lead to the worse OS (16, 17), so we compared the OS in subgroups according to the RPN from one to three. However, no significant difference of survival in all three subgroups. Interestingly, In one PRN setting, patients without PMRT has better OS than patients with PMRT: 5-year OS rates of 92.9 versus 97.2% (radio vs. nonradio, P = 0.307; **Figure 4B**). In two PRN settings, 5-year OS rates of 91.1 versus 82.6% (radio vs. nonradio, P = 0.398; **Figure 4C**), and in three PRN settings, 5-year OS rates of 90.1 versus 80.3% (radio vs. nonradio, P = 0.179; **Figure 4D**)

Then we added the tumor size and grade as a subgroup for analysis. Eventually, we found that in the setting with three PRN, The difference is fairly large between the radio and non-radio groups for patients with tumor size >14mm and grade worse than well-differentiated could benefit from the PMRT: 5-year OS rates of 90.8 versus 82.3% (radio vs. nonradio, P <0.001; **Figure 5B**).

### 3.4 Training curve and model performance

The finalized model consists of 5 hidden layers, each with 20–32–25–21–23 neurons and a dropping-out unit between them. The learning rate was 0.00701 and the dropout rate was 0.782. The training loss curves, as shown in **Figure 2**, demonstrate a gradual decrease in the loss for both the validation and training sets during the training process. However, after 96 epochs, the validation set’s loss reaches a plateau at 5.0429, while the training set’s loss continues to decrease from 5.1712. To prevent overfitting, we stop the optimization process and retain the model for testing.

In the Cox Proportional hazard model, we configure the penalizer to 0.004 and the learning rate to 0.01.

The mean of the concordance index of the deep learning algorithm is 0. 776, which is significantly higher than cox proportional hazard model (0. 641) (**Table 2**). Clearly, the performance of the deep learning model is better (0.769 vs 0.650).

**Table 2:** Performance of the survival models to predict hazard rate.

MODEL	Cross Validation	External Validation
	Concordance Index Mean	Concordance Index
Deep Learning	0.776	0.769
Cox Proportional	0.641	0.650

### 3.5 The PMRT recommender system

**Figure 6:**
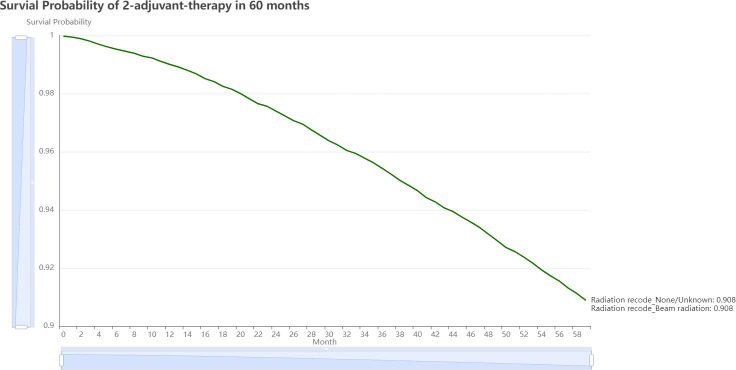
The output page of the recommender system.

Oncology professionals can input the patient’s current clinical status, including demographic, morphology, disease extent, stage, and therapy information, and submit the form for analysis. On the result page, we can view two very similar 5-year estimated survival curves for each treatment plan (**Figure 6**). The plot reveals that avoiding adjuvant radiation, which has a better chance of surviving than receiving it in the following 60 months, is the best course of treatment. This information serves as a valuable guide for oncology professionals when deciding on the most suitable adjuvant therapy strategy for individual patients. On https://github.com/snowflake-Zhao/BRCA-I-PMRT, you could find the code for this application and reproduce the performance of the model.

## 4 Discussion

This study aims to address the controversy on the potential benefit of PMRT for HR+/HER2- T1-2 N1 M0 breast cancer patients. This work found out that among HR+/HER2- T1-2 N1 M0 breast cancer patients, patients with tumor size >14mm and age >55 as well as patients with 3 PRN, tumor size >14mm and grade worse than well-differentiated could benefit from PMRT. This research not only trained an accurate deep learning model but also incorporated an explainable module to shed light on how the model predicts outcomes based on the individual feature importance. Also, we obtained the global feature importance of the model from the model and the training data. To our best knowledge, this is the first explainable recommender system to provide adjuvant treatment plan reference for HR+/HER2- T1-2 N1 M0 breast cancer patients who have undergone mastectomy.

In contrast to previous studies, such as the work by Zhao J. et al. (17), we didn’t find any statistic significance between with and without PMRT groups in all three subgroups, PRN node from one to three. Instead, we found patients not only with three PRN but also with tumor size >14mm and worse grade could benefit from PMRT. Because they only consider 143 patient, the data seems is biased so is the conclusion. However, we find out the same conclusion with Lane L. et al (18). The more PRN leads to the worse OS, so generally combining with other detrimental factors(tumor size >14mm and grade *>*grade I), the cohort could benefit from the OS. Age and grade have been similarly discussed in multiple studies as OS predictors in patients with T1T2 breast cancer and one to three PRN, their trend with OS could support our findings that tumor size >14mm and grade worse than well-differentiated could benefit from PMRT.

The determination of global feature importance is based on the model and the training dataset. Firstly, the significance of tumor size in the N1 patient population has been widely acknowledged in previous studies (10, 19, 20). Many studies classify patients into risk groups based on pT stage, with a common threshold of 2 cm in the greatest dimension (21, 22). This finding is consistent with our result, which identify tumor size as the most important feature for patient survival and PMRT decision-making. Secondly, the status of regional nodes also plays a crucial role. Existing research consistently demonstrates that patients with a single axillary lymph node (ALN) metastasis tend to have better outcomes, in terms of local regional recurrence (LRR) or OS, compared to those with two or three positive nodes (23).The cut-off values between low- and high-risk disease often fall around 20% of positive-to-dissected lymph nodes (24). Similarly, in our experiment, the number of examined and PRN are highly significant to patient survival. Furthermore, we found that patient aged between 45 to 49 years have a critical impact on the decision to receive PMRT and OS. This finding aligns with the results of Truong et al., who recommended PMRT for patients aged 45 years with 25% positive axillary nodes, medial tumor location and ER-negative in their retrospective analysis including 821 T1–2N1 breast cancer patients (25). We could conclude that age of 45 years is a critical point for T1-2 N1 HR+/HER2- breast cancer patients in PMRT decision-making. Other clinical characteristics investigated in our study did not demonstrate significant importance compared to as these four features.

To further enhance the applicability of this method in real medical settings, it is imperative to incorporate causal inference into the training and explanation processes (26–28). For instance, we could integrate causal model assumptions to enhance the interpretability of feature attributions and incorporate the causal inference ideas in designing causal models by adding sample reweighting technique into the loss function to compare the performance with our deep learning result in the future (29–32).

## 5 Conclusions

HR+/HER2- T1-2 N1M0 breast cancer patients with tumor size >14mm and age older than 54 and cohort with tumor size >14mm and grade worse than well-differentiated could benefit from the PMRT. The deep learning network performed more stably and accurately in predicting patients survival than Cox proportional hazard model on the internal test. Besides, tumor size, examined regional nodes, age at 45-49 years old and PRN are the most significant factors to the OS.

## Data availability statement

The datasets presented in this study can be found in online repositories. The names of the repository/repositories and accession number(s) can be found below: https://github.com/snowflake-Zhao/BRCA-I-PMRT.

## Ethics statement

The studies involving humans were approved by Medical Ethics Committee of Shaanxi Provincial People’s Hospital. The studies were conducted in accordance with the local legislation and institutional requirements. Written informed consent for participation was not required from the participants or the participants’ legal guardians/next of kin in accordance with the national legislation and institutional requirements.

## Author contributions

LJ: Conceptualization, Data curation, Investigation, Writing – original draft, Writing – review & editing. QZ: Conceptualization, Data curation, Formal analysis, Methodology, Project administration, Software, Validation, Visualization, Writing – original draft, Writing – review & editing. SF: Conceptualization, Data curation, Formal analysis, Methodology, Supervision, Writing – review & editing. YZ: Conceptualization, Data curation, Formal analysis, Funding acquisition, Project administration, Software, Writing – review & editing. SW: Conceptualization, Data curation, Formal analysis, Project administration, Writing – review & editing. XL: Conceptualization, Data curation, Formal analysis. FC: Conceptualization, Data curation, Formal analysis
